# Metabolic fluxes in the central carbon metabolism of *Dinoroseobacter shibae *and *Phaeobacter gallaeciensis*, two members of the marine *Roseobacter *clade

**DOI:** 10.1186/1471-2180-9-209

**Published:** 2009-09-29

**Authors:** Tobias Fürch, Matthias Preusse, Jürgen Tomasch, Hajo Zech, Irene Wagner-Döbler, Ralf Rabus, Christoph Wittmann

**Affiliations:** 1 Institute of Biochemical Engineering, Technische Universität Braunschweig, Gaußstraße 17, D-38106 Braunschweig, Germany; 2 Helmholtz Centre for Infection Research, Research Group Microbial Communication, D-38124 Braunschweig, Germany; 3 Institute for Chemistry and Biology of the Marine Environment (ICBM), University of Oldenburg, D-26111 Oldenburg, Germany

## Abstract

**Background:**

In the present work the central carbon metabolism of *Dinoroseobacter shibae *and *Phaeobacter gallaeciensis *was studied at the level of metabolic fluxes. These two strains belong to the marine *Roseobacter *clade, a dominant bacterial group in various marine habitats, and represent surface-associated, biofilm-forming growth (*P. gallaeciensis*) and symbiotic growth with eukaryotic algae (*D. shibae*). Based on information from recently sequenced genomes, a rich repertoire of pathways has been identified in the carbon core metabolism of these organisms, but little is known about the actual contribution of the various reactions *in vivo*.

**Results:**

Using ^13^C labelling techniques in specifically designed experiments, it could be shown that glucose-grown cells of *D. shibae *catabolise the carbon source exclusively via the Entner-Doudoroff pathway, whereas alternative routes of glycolysis and the pentose phosphate pathway are obviously utilised for anabolic purposes only. Enzyme assays confirmed this flux pattern and link the lack of glycolytic flux to the absence of phosphofructokinase activity. The previously suggested formation of phosphoenolpyruvate from pyruvate during mixotrophic CO_2 _assimilation was found to be inactive under the conditions studied. Moreover, it could be shown that pyruvate carboxylase is involved in CO_2 _assimilation and that the *cyclic *respiratory mode of the TCA cycle is utilised. Interestingly, the use of intracellular pathways was highly similar for *P. gallaeciensis*.

**Conclusion:**

The present study reveals the first insight into pathway utilisation within the *Roseobacter *group. Fluxes through major intracellular pathways of the central carbon metabolism, which are closely linked to the various important traits found for the *Roseobacter *clade, could be determined. The close similarity of fluxes between the two physiologically rather different species might provide the first indication of more general key properties among members of the *Roseobacter *clade which may explain their enormous success in the marine realm.

## Background

**Figure 1:**
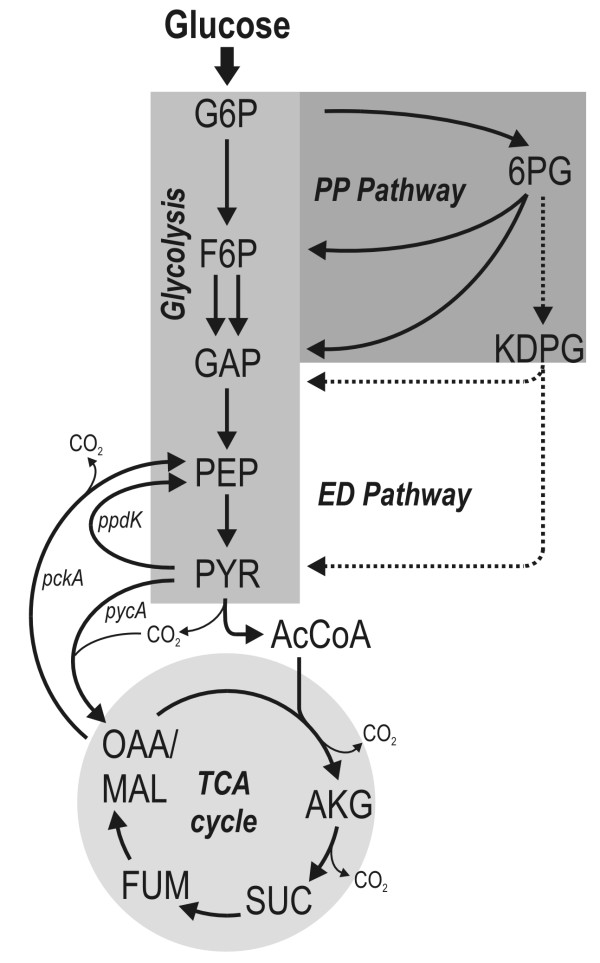
**Metabolic network of the central carbon metabolism of *Dinoroseobacter shibae ***[1]**and *Phaeobacter gallaeciensis ***[25]**as predicted from the annotated genome sequence**. G6P: glucose-6-phosphate; F6P: fructose-6-phosphate; GAP: glyceraldehyde-3-phosphate; PEP: phosphoenolpyruvate; PYR: pyruvate; AcCoA: acetyl-Coenzyme A; OGA: 2-oxoglutarate; SUC: succinate; FUM: fumarate; OAA: oxaloacetate; MAL: malate; 6PG: 6-phosphogluconate; KDGP: 2-keto-3-deoxy-6-phosphogluconate; pycA: pyruvate carboxylase; pckA: phosphoenolpyruvate carboxykinase; ppdK: pyruvate orthophosphate dikinase.

The *Roseobacter *lineage, representing a group of *Alphaproteobacteria *[1], is found in various marine habitats where it is present in high abundance, comprising up to 25% of the total bacterial community [2]. Overall, the diverse metabolic properties of the *Roseobacter *clade and its ubiquitous occurrence in marine ecosystems suggest that members of this clade play an important role in global biogeochemical processes such as cycling of carbon or sulphur [3]. Members of the *Roseobacter *clade participate in DMSP demethylation [4], the oxidation of carbon monoxide [5] and degradation of aromatic compounds [6,7]. Typically, they use external organic substrates as carbon sources [8]. Of outstanding interest is the fact that they are able to generate energy from light (aerobic anoxygenic phototrophy) [9] and thus contribute significantly to phototrophic energy generation [10,11]. All these important traits are linked to the core part of central carbon metabolism involved in the breakdown of nutrients and the supply of metabolites and energy for various cellular requirements. Recent efforts in genome sequencing and annotation of *Roseobacter *members have provided a first insight into the repertoire of underlying metabolic reactions available (Figure 1) and have led to different suggestions for possible pathways that might be involved in important physiological functions [12]. As an example, a mixotrophic CO_2 _assimilation pathway has been proposed for *R. denitrificans*, in which CO_2 _is fixed either (i) via the combined action of pyruvate-orthophosphate dikinase and phosphoenolpyruvate carboxylase or (ii) via pyruvate carboxylase [13]. For glucose catabolism, up to three alternative routes are encoded in the genome: glycolysis, the pentose phosphate pathway and the Entner-Doudoroff pathway. At this point, it seems highly relevant to study the contribution of these potential pathways to the metabolism of bacteria in the *Roseobacter *clade to improve our understanding of their physiology. Our current knowledge of the *in vivo *fluxes through intracellular pathways among the *Roseobacter *lineage is still very limited.

To address this issue, we applied metabolic flux analysis using ^13^C labelled isotopes to gain a first insight into the central catabolic pathways of *Dinoroseobacter shibae *DFL12 [1] and *Phaeobacter gallaeciensis *DSM 17395 [14]. These species represent two prominent members of the *Roseobacter *clade. *P. gallaeciensis *has received strong interest due to its ability to produce the antibiotic tropodithietic acid. *D. shibae *was isolated as a novel species from marine dinoflagellates and lives in a symbiotic relationship with eukaryotic algae [15]. Metabolic flux analysis using ^13^C labelled isotopes has proven a key technology in the unravelling of metabolic pathways and has recently been used to study different microorganisms mainly linked to biotechnological production processes [16-19]. No such study has yet been performed for members of the *Roseobacter *clade.

## Results and Discussion

### Cultivation profile

**Figure 2:**
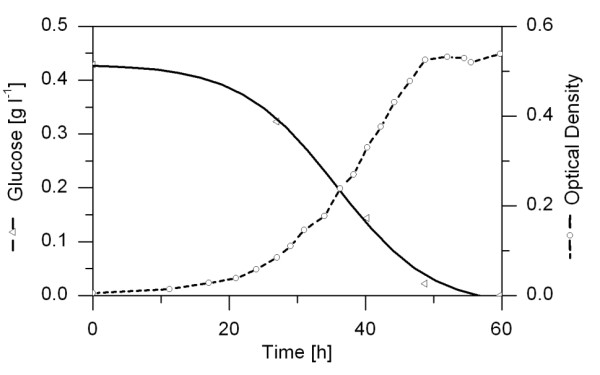
**Time courses of glucose concentration and optical density during a batch cultivation of *D. shibae *in shake flasks under constant light**.

The cultivation profile of *D. shibae *on defined medium with glucose as the sole carbon source is displayed in Figure 2. After an initial adaptation phase, cells grew exponentially with a constant specific growth rate of 0.11 h^-1^. After 50 hours of cultivation the carbon source was depleted and cells entered a stationary phase. The biomass yield was 0.45 g cell dry mass per g glucose consumed, indicating efficient utilisation of the carbon source for growth. A similar growth profile was determined for *P. gallaeciensis*.

### Pathways for glucose catabolism

**Figure 3:**
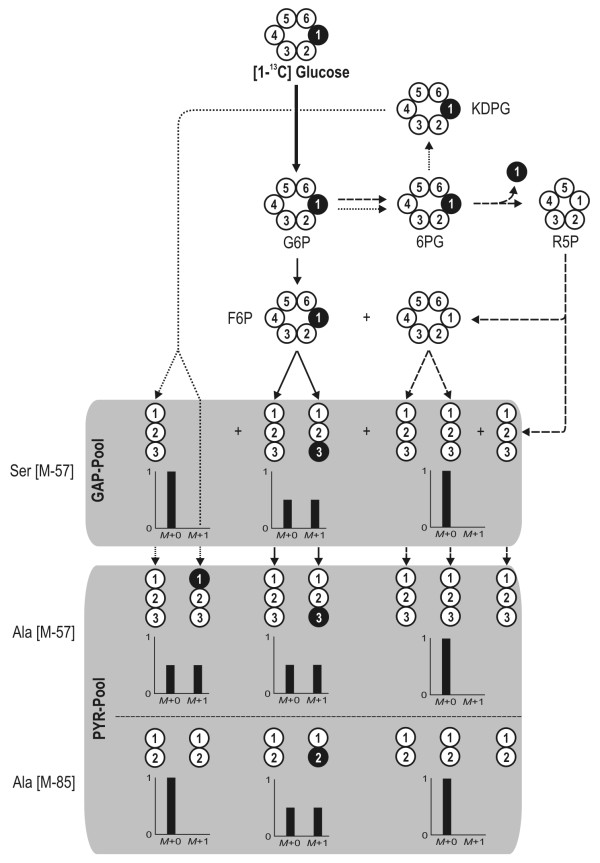
**Theoretical labelling pattern of the C_3 _pool (GAP and PYR) derived from 99% [1-^13^C] glucose depending on activities in the carbon core metabolism**. The three major routes for glucose catabolism are presented: glycolysis (solid lines), the pentose phosphate pathway (dashed lines) and the Entner-Doudoroff pathway (dotted lines). White circles represent unlabelled (^12^C) carbon whereas black circles indicate labelled (^13^C) carbon. The numbers reflect the position of the carbon atom within the molecule. Ala: alanine; G6P: glucose 6-phosphate; 6PG: 6-phosphogluconate; KDPG: 2-keto-3-deoxy-6-phosphogluconate; F6P: fructose 6-phosphate; R5P: ribose 5-phosphate; GAP: glyceraldehyde 3-phosphate; PYR: pyruvate.

The carbon core metabolism of *D. shibae *and *P. gallaeciensis *consists of three potential routes for glucose catabolism. Glucose can be alternatively catabolised via glycolysis (EMP), the pentose phosphate pathway (PPP) and the Entner-Doudoroff pathway (EDP). The use of [1-^13^C] glucose by each individual pathway leads to a different labelling pattern in specific fragments of alanine and serine, which can be taken as a clear differentiation of flux (Figure 3). For *D. shibae *the corresponding [M-57] fragment of serine did not show any enrichment of ^13^C but rather reflected the pattern resulting from the natural abundance of ^13^C only (Table 1). Any contribution of glycolysis to formation of this metabolite and its precursor 3-phosphoglycerate can therefore be excluded as this would lead to enrichment of ^13^C at the C_3 _position, yielding a higher fraction of *M*+1 labelled molecules of Ser. Thus glycolytic flux obviously was not present. The two remaining possibilities, the PPP and the ED pathway, can be differentiated by the labelling pattern of alanine, which represents the pyruvate pool in the cell. The high enrichment of ^13^C label in the [M-57] fragment of alanine indicates a large contribution of the ED pathway, since formation via the PPP would lead to non-labelled alanine. The [M-85] fragment of alanine, comprising only the carbon atoms C_2 _and C_3 _of pyruvate, was not enriched in ^13^C, showing that pyruvate was labelled only at its C_1 _position. This perfectly matches the isotope pattern expected for the ED pathway, whereas glycolytic flux would have resulted in label enrichment at the C_3 _position, and further confirms the flux distribution.

**Table 1:** Selected TBDMS^a^-amino acid fragments used in the study derived from *D. shibae *and *P. gallaeciensis *grown on 99% [1-^13^C] glucose.

	Fragment	C-Atoms	Mass isotopomer distribution (% of total pool)							
			
			*D. shibae*				*P. gallaeciensis*			
				
			*M*+0	*M*+1	*M*+2	*M*+3	*M*+0	*M*+1	*M*+2	*M*+3
Ala	M-57	1-3	50.0 ± 0.2	48.2 ± 0.2	1.8 ± 0.0	0.01 ± 0.01	49.2 ± 0.0	49.3 ± 0.0	1.5 ± 0.0	0.0 ± 0.0
	M-85	2-3	96.8 ± 0.1	3.2 ± 0.1	0.0 ± 0.0		97.2 ± 0.0	2.8 ± 0.0	0.0 ± 0.0	
	f302	1-2	51.2 ± 0.1	48.2 ± 0.1	0.6 ± 0.0		50.1 ± 0.1	49.3 ± 0.1	0.6 ± 0.0	
Asp	M-57	1-4	72.4 ± 0.7	23.2 ± 0.5	4.3 ± 0.2	0.12 ± 0.01	64.2 ± 0.2	29.4 ± 0.1	6.2 ± 0.2	0.13 ± 0.07
	M-85	2-4	83.3 ± 0.6	16.2 ± 0.6	0.4 ± 0.1	0.10 ± 0.03	80.0 ± 0.1	19.4 ± 0.0	0.6 ± 0.0	0.04 ± 0.02
	f302	1-2	82.1 ± 0.3	17.6 ± 0.3	0.2 ± 0.0		76.3 ± 0.1	23.5 ± 0.0	0.3 ± 0.1	
Glu	M-57	1-5	80.7 ± 0.3	18.4 ± 0.4	0.8 ± 0.1	0.05 ± 0.03	78.1 ± 0.5	20.8 ± 0.3	0.9 ± 0.2	0.09 ± 0.03
	M-85	2-5	92.1 ± 0.2	7.5 ± 0.2	0.3 ± 0.0	0.06 ± 0.00	93.6 ± 0.1	6.2 ± 0.1	0.0 ± 0.0	0.09 ± 0.01
	f302	1-2	83.4 ± 0.2	16.2 ± 0.2	0.3 ± 0.0		81.2 ± 0.3	18.4 ± 0.1	0.4 ± 0.2	
Gly	M-57	1-2	96.1 ± 0.0	3.8 ± 0.0	0.1 ± 0.0		97.2 ± 0.1	2.8 ± 0.1	0.03 ± 0.02	
	M-85	2	98.8 ± 0.1	1.1 ± 0.0			99.0 ± 0.0	0.9 ± 0.0		
Phe	M-57	1-9	85.7 ± 0.6	13.0 ± 0.6	0.6 ± 0.1	0.08 ± 0.03	86.7 ± 0.9	11.6 ± 0.3	0.5 ± 0.1	0.02 ± 0.01
	f302	1-2	95.9 ± 0.3	4.1 ± 0.3	0.0 ± 0.0		96.7 ± 0.2	3.3 ± 0.2	0.0 ± 0.0	
Ser	M-57	1-3	95.3 ± 0.3	4.6 ± 0.3	0.0 ± 0.0	0.07 ± 0.03	96.7 ± 0.1	3.3 ± 0.1	0.0 ± 0.0	0.09 ± 0.02
	M-85	2-3	97.7 ± 0.1	2.3 ± 0.1	0.0 ± 0.0		98.0 ± 0.1	2.0 ± 0.1	0.0 ± 0.0	
	f302	1-2	95.6 ± 0.0	3.9 ± 0.0	0.5 ± 0.0		96.8 ± 0.1	2.8 ± 0.0	0.4 ± 0.0	
Tyr	M-57	1-9	86.2 ± 0.7	12.8 ± 0.1	0.5 ± 0.3	0.06 ± 0.09	87.7 ± 0.2	11.4 ± 0.4	0.5 ± 0.0	0.08 ± 0.06
	f302	1-2	96.1 ± 0.2	3.9 ± 0.2	0.0 ± 0.0		97.3 ± 0.4	2.7 ± 0.4	0.0 ± 0.0	

^*a *^*tert*-butyldimethylsilyl; fragmentation patterns are described elsewhere [27]

**Figure 4:**
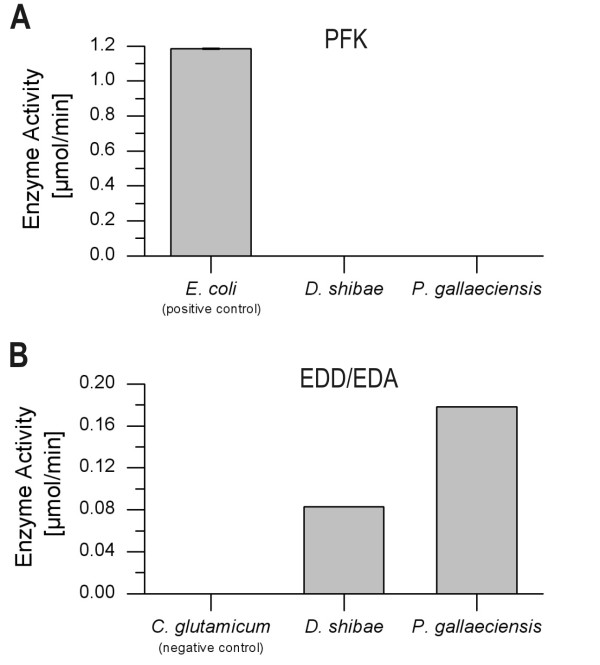
**In vitro activities of key enzymes of the different catabolic pathways for D. shibae and P. gallaeciensis**. PFK: 6-phosphofructokinase; EDD: 6-phosphogluconate dehydrogenase; EDA: 2-keto-3-deoxy-6-phosphogluconate aldolase.

Interestingly, *P. gallaeciensis *showed almost identical characteristics and obviously also uses mainly the ED pathway during growth on glucose. The quantification of relative flux (Eqs. 2 and 3) revealed that the use of the ED pathway amounts to >99%, whereas glycolysis and PPP contribute only <1% (Table 2). Compared to other microorganisms such as *E. coli *[20], *B. subtilis *[21], *B. megaterium *[18] or *C. glutamicum *[22] grown on glucose, this is a rather unusual flux pattern. Most organisms use glycolysis and the pentose phosphate pathway concomitantly but at varying ratios (Table 2). Exclusive utilisation of the ED pathway, as found here, has been previously observed in selected species of *Pseudomonas *or *Arthrobacter *where this behaviour was attributed to a lack of phosphofructokinase [23,24]. Among the two microorganisms studied, *D. shibae *does contain a gene encoding for this enzyme, whereas *P. gallaeciensis *does not. For both *Roseobacter *species, in contrast to *E. coli *as positive control, phosphofructokinase activity could not be detected, clearly explaining the lack of glycolytic flux (Figure 4B). While this matches with the genomic repertoire of *P. gallaeciensis*, we conclude at this stage that the phosphofructokinase in *D. shibae *is either not expressed, might have another function or even is a non-functional protein. The flux pattern for both organisms is supported by enzymatic assays showing high *in vitro *activity of 6-phosphogluconate dehydratase and 2-dehydro-3-deoxyphosphogluconate aldolase, the two key enzymes in the Entner-Doudoroff pathway (Figure 4A).

**Table 2:** Comparison of catabolic pathway activity and origins of metabolic intermediates in central carbon metabolism of *D. shibae*, *P. gallaeciensis *and other bacteria derived from carbon labelling experiments.

	Pathway activity/Fractional pool composition [%]					
	
	*D. shibae*^*a*^	*P. gallaeciensis*^*a*^	***B. subtilis ***[21]	***B. megaterium ***[18]	***C. glutamicum ***[35]	***E. coli ***[20]
Glycolysis	< 1	< 1	27	46	49	73
PPP	< 1	< 1	72	49	48	22
ED pathway	> 99	> 99	n.a.	n.a.	n.a.	4

PEP from PYR	0	0	0	0	0	0
PEP from OAA	0	0	14	0	16	0

^*a*^this study

n.a. = not available in the organism

### Pathways for PEP synthesis - contribution of pyruvate-orthophosphate dikinase and phosphoenolpyruvate carboxykinase

Based on the labelling data given above, the formation of PEP from pyruvate by pyruvate-orthophosphate dikinase or via pyruvate carboxylase and phosphoenolpyruvate carboxykinase would result in the presence of PEP with^13^C enrichment at position C_1_. However, the [f302] fragments of Phe and Tyr, each corresponding to the carbon atoms C_1 _and C_2 _of PEP, do not show significant enrichment of ^13^C (Table 1). The same holds for the [M-57] fragment, which corresponds to the entire carbon skeleton of Phe and Tyr and thus all precursors, that is, PEP and E4P. Flux quantification using Equations 4 and 5 confirms that PEP is solely synthesised by the reactions of lower glycolysis (Table 2). This is an interesting finding with respect to the recently suggested mixotrophic CO_2 _assimilation pathway for some members of the *Roseobacter *clade, which also involves the potential contribution of pyruvate orthophosphate dikinase (PPDK) [13]. Despite the putative gene for this protein also being annotated for the species investigated here, we could clearly demonstrate that the formation of PEP from PYR is not active *in vivo *under the conditions studied.

### Pathways for oxaloacetate synthesis - contribution of CO_2 _assimilation and oxidative TCA cycle

**Figure 5:**
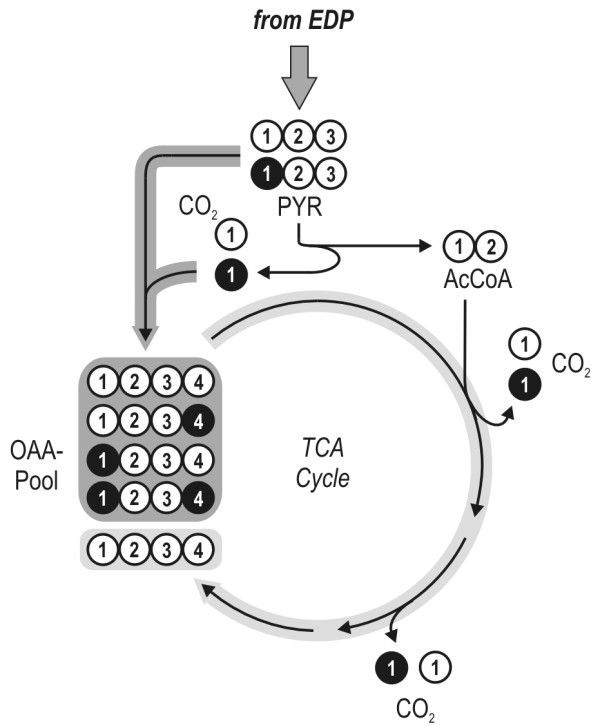
**Schematic overview of the two major pathways from pyruvate towards oxaloacetate**: (i) direct carboxylation via pyruvate carboxylase or (ii) pyruvate dehydrogenase and the energy forming reactions of the TCA cycle. The conserved carbon transfer of the underlying reactions yields a specific labelling pattern for oxaloacetate formed by each pathway which is presented in this figure. White circles represent ^12^C whereas black circles indicate labelled ^13^C. The numbers given reflect the position of the carbon atom within the molecule. AcCoA: acetyl-Coenzyme A; EDP: Entner-Doudoroff pathway; OAA: oxaloacetate; PYR: pyruvate; TCA: tricarboxylic acid.

Oxaloacetate as a central metabolite can be formed by two major pathways, that is, carboxylation involving pyruvate carboxylase or via pyruvate dehydrogenase and the energy-generating reactions of the TCA cycle. The following data clearly suggest that both pathways are active simultaneously in the two Roseobacters. For the experimental setup chosen and carbon transfer in the underlying metabolic reactions, the carboxylation of pyruvate is the only reaction that leads to ^13^C labelled oxaloacetate (Figure 5). The label can be present in carbon positions C_1 _or C_4_, whereby single- or double-labelled molecules can be formed, depending on the incorporation of ^12^CO_2 _or ^13^CO_2_. In contrast, the alternative route via the cyclic respiratory mode of the TCA cycle yields exclusively non-labelled oxaloacetate. In all possible cases the labelled carbon atoms from either pyruvate or oxaloacetate are released in the decarboxylation steps of the TCA cycle as ^13^CO_2_. Inspection of the labelling pattern of aspartate, corresponding to the oxaloacetate backbone, immediately shows that single- and double-labelled mass isotopomers are present in significant amounts for *D. shibae *and *P. gallaeciensis*, indicating *in vivo *activity of pyruvate carboxylase in both strains (Table 1). However, the relative fractions of these ^13^C enriched mass isotopomers are relatively small, excluding sole contribution of this reaction to oxaloacetate synthesis. The dominant fraction consists of non-labelled molecules, obviously derived via the oxidative TCA cycle. We thus conclude that the *cyclic *respiratory mode of the TCA cycle is active *in vivo *in both strains. For *D. shibae*, which possesses a photosystem for energy generation, this mode might display an important strategy to derive energy under conditions where the photosystem is not active, for example, during the night or in deeper water regions.

## Conclusion

Being one of the first metabolic studies of members of the *Roseobacter *clade using the ^13^C labelling experimental approach, a deeper insight into the activity of the important metabolic routes of *D. shibae *and *P. gallaeciensis *was achieved. Interestingly, the use of intracellular pathways is highly similar in the studied species *D. shibae *and *P. gallaeciensis*. This stands in surprising contrast to the overall differences in phenotypic behaviour exhibited by these two strains, since *D. shibae *is an algal-associated microorganism whereas *P. gallaeciensis *is free-living in marine habitats. However, this may be a first indication of more general key properties among members of the *Roseobacter *clade that explain their enormous success in the marine realm.

## Methods

### Strains, medium and growth conditions

The strains used in this study are the genome sequenced strains *Dinoroseobacter shibae *DFL12 [1] and *Phaeobacter gallaeciensis *DSM 17395 [14]. For cultivation of both strains a defined, synthetic seawater medium (minimal medium) was used [25], containing the following components per litre of medium: 4.0 g NaSO_4_, 0.2 g KH_2_PO_4_, 0.25 g NH_4_Cl, 20.0 g NaCl, 3.0 g MgCl_2_·6 H_2_O, 0.5 g KCl and 0.15 g CaCl_2_·2 H_2_O, 0.19 g NaHCO_3_, 1 ml trace element solution and 10 ml vitamin solution. The final glucose concentration in the medium was in the range of 0.4 to 0.9 g l^-1^. The trace element solution contained 2.1 g Fe(SO_4_)·7 H_2_O, 13 ml 25% (v/v) HCl, 5.2 g Na_2_EDTA·2 H_2_O, 30 mg H_3_BO_3_, 0.1 g MnCl_2_·4 H_2_O, 0.19 g CoCl_2_·6 H_2_O, 2 mg CuCl_2_·2 H_2_O, 0.144 g ZnSO_4_·7 H_2_O and 36 mg Na_2_MoO_4_·2 H_2_O per litre. The vitamin solution for *D. shibae *contained the following components per litre: 0.2 g biotin, 2.0 g nicotinic acid and 0.8 g 4-aminobenzoic acid. All solutions were sterilised separately and mixed at room temperature prior to inoculation. For carbon labelling experiments 99% [1-^13^C] glucose (Euriso-Top, Saint-Aubin, France) was used as substrate. The cultivations were carried out on orbital shakers at 200 rpm in 500 ml shaken flasks with a culture volume of 50 ml at 37°C (*D. shibae*) and 28°C (*P. gallaeciensis*). To ensure comparable conditions between the two microorganisms and avoid any potential influencing effects of phototrophy in *D. shibae*, both organisms were cultivated in the light. Under these conditions, no bacteriochlorophyll is synthesised *D. shibae *[1] and therefore no active photosystem is present that might affect energy metabolism and thus induce changes in the fluxes through the main metabolic pathways.

### Analytics

Cell concentration was monitored by measuring the optical density (OD) at 600 nm or by gravimetry [26]. The ^13^C labelling pattern of the amino acids contained in the cell protein was determined as follows [27]. Cells were harvested during exponential growth phase at half-maximal optical density including a washing step in 0.9% NaCl solution, followed by lyophilisation. Subsequently, 4 mg of lyophilised cells was resuspended in 200 μl of 6 M HCl and incubated at 110°C for 24 h. The obtained hydrolysate was neutralised by addition of 6 M NaOH and cleared of insoluble matter (0.2 μm centrifugal filter device Ultrafree MC, Millipore, Bedford, MA, USA). Subsequently, 50 μl of the hydrolysate was transferred to a 2 ml sample vial, lyophilised and derivatised at 80°C for 60 min with 50 μl N, N-dimethylformamide (Carl Roth, Karslruhe, Germany) containing 0.1% (v/v) pyridine and 50 μL N-methyl-*tert*-butyldimethylsilyl-trifluoroacetamide (MBDSTFA, Macherey-Nagel, USA). GC/MS measurements were carried out as described earlier [27]. Subsequent MS data processing was carried out according to Fürch et *al*. [18] and Lee et *al*. [28,29].

### Preparation of cell extracts for enzyme activity measurements

Cells were harvested by centrifugation at 10,000 g for 10 min, washed twice with 100 mM Tris-HCl (pH 7.0) containing 20 mM KCl, 5 mM MnSO_4_, 2 mM DTT and 0.1 mM EDTA, and then resuspended in the same buffer. Afterwards the cells were disrupted by sonification for 1 min using an ultrasonic disrupter (Sonifier W250 D, Branson, Danbury, USA) with an amplitude of 30%. Cell debris was removed by centrifugation. The resulting crude cell extract was immediately used to determine specific enzyme activity. All operations were carried out on ice.

#### Enzyme assays

Enzyme activities in crude cell extract were measured spectrophotometrically. All compounds of the reaction mixture were pipetted into a cuvette with a 1 cm light path and reactions were initiated by adding the cell extract or substrate respectively. The total protein concentration of the crude cell extract was determined using RotiQuant (Carl Roth GmbH, Karlsruhe, Germany). The overall activity of 6-phosphogluconate dehydratase (EDD) and 2-dehydro-3-deoxyphosphogluconate aldolase (EDA) was measured using a two-step reaction [30]. For this purpose 0.8 μmol 6-phosphogluconate, 1 μmol MgCl, 5 μmol Tris-HCl buffer (pH 7.65) and 100 μl of extract were incubated in a total volume of 0.5 ml for 5 min at room temperature. The reaction was stopped by dilution with 2 ml of the same buffer and then by heating in a boiling water bath for 2 min. After centrifugation, the supernatant solution was assayed for pyruvate with NADH and lactate dehydrogenase according to Peng and Shimizu [31]. The activity of 6-phosphofructosekinase (PFK) in the crude cell extract was assayed as described by Gancedo and Gancedo [32]. The reaction mix contained 50 mM imidazole HCl (pH 7.0), 0.05 mM ATP, 5 mM MgCl_2_, 1 mM EDTA, 0.25 mM NADH, 0.25 mM fructose 6-phosphate (F6P), 0.5 U aldolase, 0.5 U glycerolphosphate dehydrogenase and 0.5 U triosephosphate isomerase.

### Metabolic flux calculations

Metabolic flux calculations were performed as described previously [18]. Briefly, metabolic flux ratio analysis was used to gain information about the flux distribution at important branch points within the network. As several alternative pathways may lead to a particular product, the fractional contribution (metabolic flux ratio) of each pathway was determined based on the molecular mass distributions of the reactants and the product according to Fischer and Sauer [33]. For the performed calculations, corrected mass spectra of selected fragments of serine, glycine, alanine, phenylalanine, tyrosine, aspartate and glutamate were used in this study (see Table 1). As the amino acids are synthesised from precursor metabolites of the central carbon metabolism with a known and well conserved carbon transition, their labelling pattern can be used to conclude the corresponding labelling pattern of their precursors [34]. To gain important information about the position of the labelling within the molecule, different fragments were considered simultaneously. In general, TBDMS-derivatised amino acids yield characteristic fragments by electron impact ionisation. The [M-57] fragment of each amino acid contains the complete carbon backbone, whereas the [M-85] fragment lacks the carbon at the C_1 _position that corresponds to the carbon atom of the carboxyl group of the amino acid. The third fragment considered - [f302] - always contains the C_1 _and C_2 _carbon of the corresponding amino acid.

In the case of alternative pathways yielding a specific product, the fractional contribution of each pathway can be determined concerning the mass distributions of the reactants and the product according to Eq. (1) [33].(1)

In Eq. (1) index *X *indicates the product molecule whereas the consecutive numbers *1 *through *n *represent reactant molecules of alternative pathways contributing to the mass distribution of the product pool. The corresponding fractional amount of each pathway *f *can then be calculated by considering two additional constraints: (i) all fractions must have a positive value and (ii) their sum has to equal 1. A more detailed description will be given in the following respective sections.

### Theoretical framework for flux estimation

To carry out metabolic flux calculations for *D. shibae *and *P. gallaeciensis*, a metabolic network was constructed based on genome data (GenBank accession numbers NC_009952 [*D. shibae*] and NZ_ABIF00000000 [*P. gallaeciensis*]). As we focused on the central carbon metabolism, the major catabolic routes for glucose as well as the reactions linking the C_3 _and C_4 _pools were considered. In terms of glucose catabolism, the annotated genome revealed the presence of the genes encoding for glycolytic enzymes, enzymes of reactions in both the PPP and the ED pathway and TCA cycle. For *D. shibae*, pyruvate carboxylase (PYRCx) and phosphoenolpyruvate carboxykinase (PEPCk) were found to be the interconnecting reactions between the C_3 _and the C_4 _pool. Furthermore, a gene encoding for pyruvate orthophosphate dikinase (PPDK) is annotated, indicating a potential exchange flux between the PYR and PEP pool. A summary of all reactions considered is presented in Figure 1. To resolve the metabolic fluxes through catabolic pathways and around important branch points within the metabolic network, appropriate approaches involving the mass patterns of different amino acid fragments were developed.

### Strategy for the estimation of glucose catabolic fluxes

**Figure 6:**
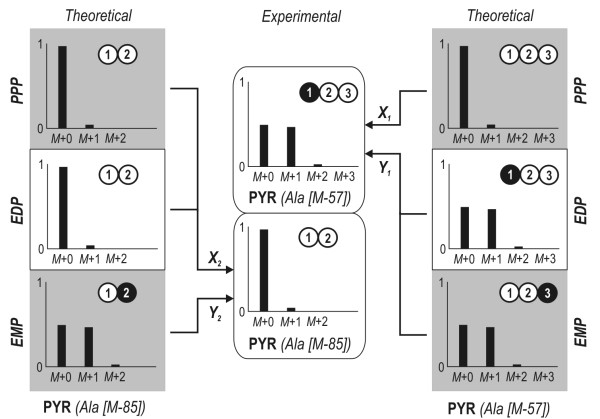
**Strategy to estimate relative flux through major catabolic pathways**. To completely resolve the contribution of each route, theoretical mass distributions of the [M-57] and [M-85] fragments of alanine were compared to the experimental data. In this schematic illustration, white circles represent unlabelled (^12^C) carbon whereas black circles indicate labelled (^13^C) carbon. The numbers given reflect the position of the carbon atom within the molecule. EDP: Entner-Doudoroff pathway; EMP: Embden-Meyerhof-Parnas pathway; PPP: pentose phosphate pathway.

In Figure 3 the theoretical labelling patterns of the C_3 _pool depending on the activity of the glycolysis, PPP and ED pathways are presented. It can be taken from the illustration that the combined analysis of two fragments derived from PYR (Ala [M-57] and Ala [M-85]) enables the contributions of each pathway to be resolved. The scheme for the estimation of the major catabolic pathways is shown in Figure 6. A comparison of the theoretical mass distribution pattern of the Ala [M-57] fragment derived from the activity of each pathway and the experimental data allows differentiation between the activity of the PPP and the combined flux through EMP and EDP (Eq. 2). The latter cannot be further subdivided as the resulting mass patterns for Ala [M-57] are similar for both pathways. The Ala [M-85] fragment therefore provides additional information for complete resolution of the three catabolic pathways. Its theoretical mass distribution compared to the experimental data yields the activity of the EMP pathway and the combined flux through EDP and PPP (Eq. 3).(2)

### Strategy for estimating fluxes around the PEP pool

**Figure 7:**
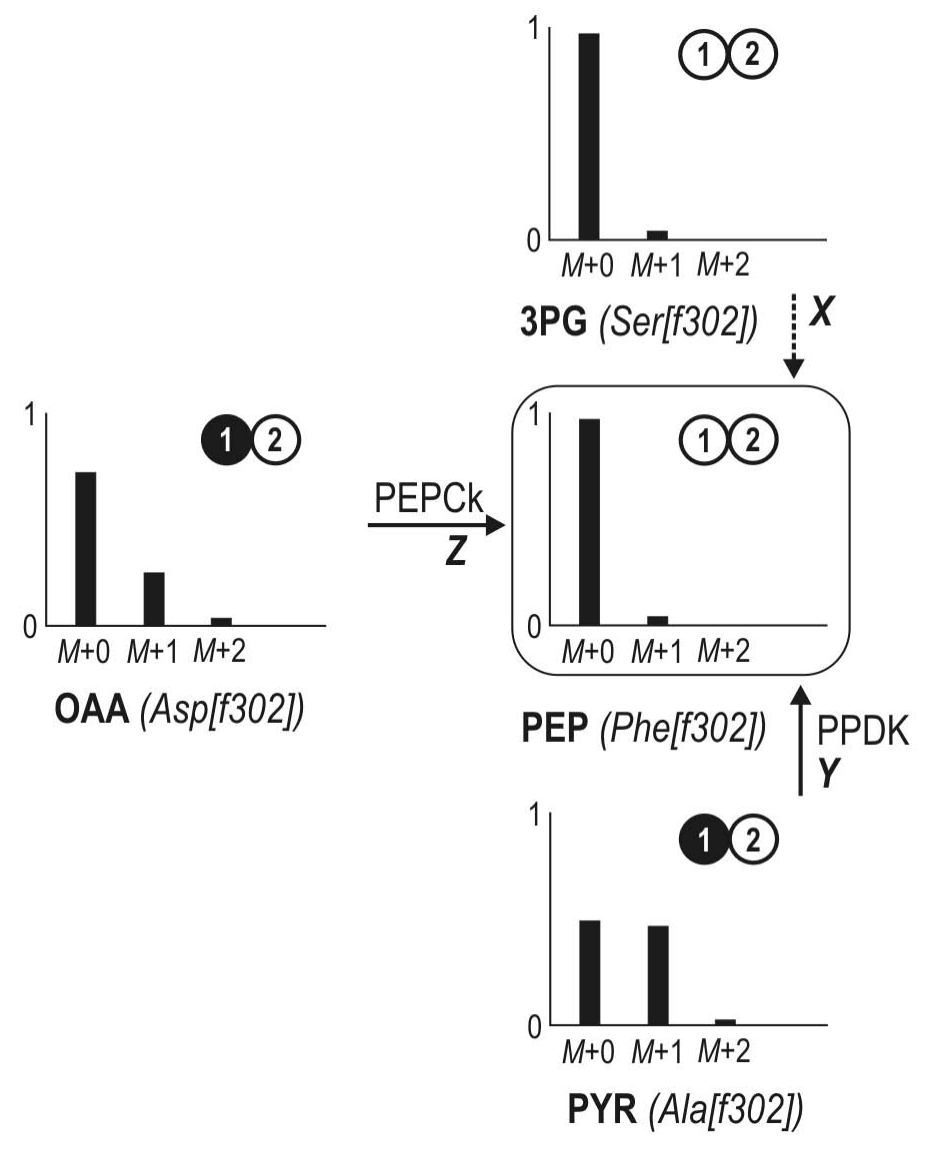
**Estimation of fluxes into the PEP pool**. The fractional contribution of each alternative pathway (*X*, *Y*, *Z*) directly follows from the mass pattern of the [f302] fragments of the precursors 3-phosphoglycerate (serine), oxaloacetate (aspartate), pyruvate (alanine) and the product PEP (phenylalanine). The corresponding isotopomer of each molecule is illustrated next to the experimental data (mass spectra). White circles represent ^12^C whereas black circles indicate labelled ^13^C. The numbers given reflect the position of the carbon atom within the molecule. PEPCk: phosphoenolpyruvate carboxykinase; PPDK: pyruvate-orthophosphate dikinase.

The metabolic reaction network around the PEP node is presented in Figure 7. It contains all reactions for which the corresponding genes have been annotated in the KEGG database. The pathways through lower glycolysis and the reactions catalysed by phosphoenolpyruvate carboxykinase (PEPCk) and pyruvate orthophosphate dikinase (PPDK) yielding PEP from either OAA or PYR are considered. Fluxes into the PEP pool were resolved using the mass distribution patterns of the [f302] fragments (carbon atoms at position C_1 _and C_2_) of the amino acids directly connected to the PEP pool according to Equations 4 and 5.(4)

## Abbreviations

[1-^13^C] glucose: Glucose labelled at C1-position; 3PG: 3-phosphoglycerate; 6PG: 6-phosphogluconate; AcCoA: Acetyl-Coenzyme A; Ala: Alanine; CLE: labelling experiment; EDA: 2-keto-3-deoxy-6-phosphogluconate aldolase; EDD: 6-phosphogluconate dehydrogenase; EDP: Entner-Doudoroff pathway; EMP: Embden-Meyerhof-Parnas; F6P: Fructose-6-phosphate; FUM: Fumarate; G6P: Glucose 6-phosphate; GAP: Glyceraldehyde 3-phosphate; GC/MS: Gas chromatography/mass spectrometry; KDPG: 2-keto-3-deoxy-6-phosphogluconate; MAL: Malate; MDV: Mass distribution vector; OAA: Oxaloacetate; OD: Optical density; OGA: 2-oxoglutarate; PEP: Phosphoenolpyruvate; PEPCk, pckA: Phosphoenolpyruvate carboxykinase; PFK: 6-phosphofructokinase; Phe: Phenylalanine; PPP: Pentose phosphate pathway; PPDK: ppdK Pyruvate orthophosphate dikinase; PYR: Pyruvate; PYRCx, pycA: Pyruvate carboxylase; R5P: Ribose 5-phosphate; Ser: Serine; SUC: Succinate; TCA: Tricarboxylic acid; Tyr: Tyrosine

## Authors' contributions

TF carried out the labelling analytics and data processing, performed the flux calculations and drafted the manuscript together with CW. MP performed the cultivation experiments for *D. shibae*. HZ performed the cultivation experiments for *P. gallaeciensis*. JT assisted in method set-up for cultivation and analytics. IWD helped to draft the manuscript. RR helped to draft the manuscript. CW conceived, designed and coordinated the study and drafted the manuscript together with TF. All authors read and approved the final manuscript.
